# Thumb-Sparing Surgery for Digital Papillary Adenocarcinoma: A Case Report

**DOI:** 10.7759/cureus.59132

**Published:** 2024-04-27

**Authors:** Nikita Golovachev, Kassem Ghayyad, Ryan Durfee, Abdo Bachoura

**Affiliations:** 1 Orthopedic Surgery, Rothman Orthopaedics Florida at AdventHealth, Orlando, USA

**Keywords:** wide local excision, thumb-sparing surgery, thumb reconstruction, hand tumor, digital papillary carcinoma

## Abstract

Digital papillary adenocarcinoma (DPA) is a rare eccrine sweat gland tumor that often appears as a solitary, non-painful, gradually enlarging mass. Clinically, DPA presents considerable challenges due to its high likelihood of recurrence and its tendency to spread to the lymph nodes and lungs. This case report focuses on the surgical treatment of a unique case of DPA located on the dorsal thumb in a 46-year-old male. The patient initially underwent wide local excision with temporary wound coverage, and once final histopathological findings confirmed negative margins, a second procedure consisting of thumb interphalangeal joint fusion and first dorsal metacarpal artery flap coverage was performed. Eighteen months later, the patient continued to work in landscaping, performing the physically demanding tasks required by the job. This case demonstrates the feasibility of thumb preservation in the setting of soft tissue malignancy once negative margins are obtained.

## Introduction

Digital papillary adenocarcinoma (DPA) is an extremely rare eccrine sweat gland malignancy, typically manifesting as a single, painless mass with slow growth [1]. Demographic data indicate that DPA predominantly affects Caucasian males, typically in their middle to later adult years [2,3], and has an incidence of 0.08 per 1,000,000 person-years [4]. It can manifest in solid or cystic forms and is typically found on the volar surfaces of the fingers and toes, potentially spreading to the adjacent skin [2,5]. DPA poses significant challenges in clinical management due to its high local recurrence rate, up to 50%, and its propensity to metastasize, most frequently affecting the lymph nodes and lungs [3]. In a series of patients, a sentinel lymph node (SLN) biopsy revealed metastatic disease in 17% of patients with DPA [6].

Current recommendations for treating DPA involve the surgical removal of the tumor through wide local excision or, in some cases, amputating the affected digit [4,7]. Additionally, it's advised to monitor patients over a long period for any signs of local recurrence or metastatic spread [1]. In one study, among patients who experienced a recurrence of DPA after initial excision, only 5% of those who underwent secondary surgery (either re-excision or digital amputation) within six months of the initial excision experienced a second recurrence [3]. In contrast, 50% of patients who did not undergo subsequent re-excision or amputation within the same timeframe experienced a recurrence [3].

This report describes a two-stage treatment strategy that involved an initial wide local excision to obtain negative margins and a subsequent thumb-sparing reconstructive procedure, as opposed to thumb amputation for the treatment of a patient with DPA.

## Case presentation

A 46-year-old right-hand dominant male patient presented with a rapidly enlarging mass on his left thumb over the past 18 months. He first noticed this mass as a small wart-like growth eight years prior. The mass steadily enlarged, becoming a major concern for the patient. Despite its growth, the patient reported no pain or discomfort. He had previously attempted to lance and drain the mass but was unable to express any contents. There was no history of trauma or previous medical interventions for this condition. During his work as a landscaper, he reported frequent punctures from various palms and thorns. The patient did not report unusual fevers, chills, or weight loss and had no personal history of cancer. Family history included maternal ovarian and rectal cancer.

**Figure 1:**
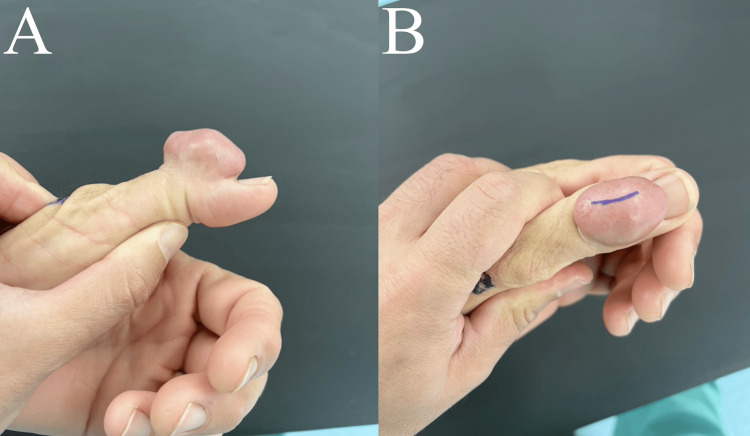
Clinical photographs The left dorsal thumb mass had a soft consistency when palpated.

**Figure 2:**
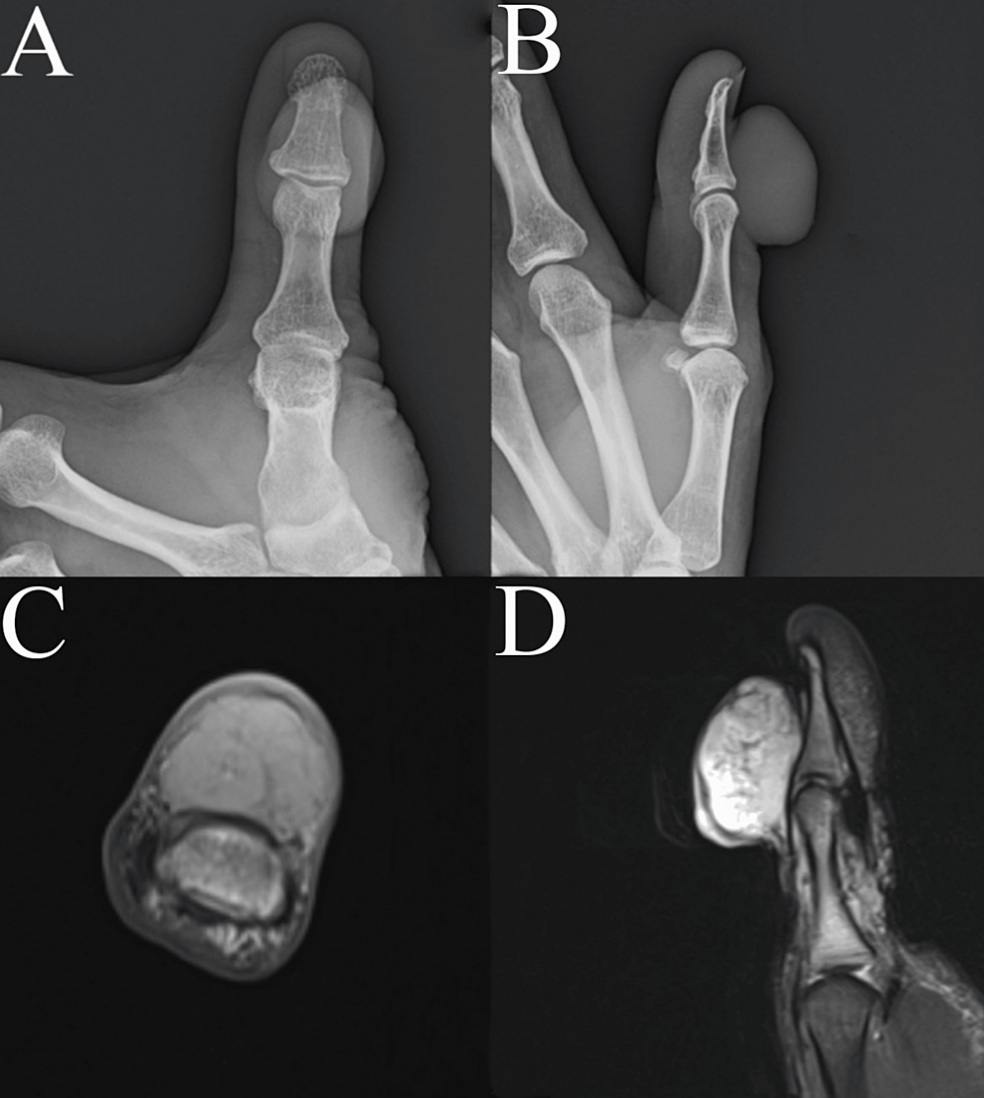
Imaging test results A: Posteroanterior; B: Lateral X-ray of the left thumb; C: Axial; D: Sagittal T2 fat suppression MRI of the left thumb showing an exophytic mass centered at the level of the interphalangeal joint.

On physical examination, the mass was pedunculated, non-tender, and located on the dorsal aspect of the thumb, measuring 4 x 3 x 2 cm (Figure 1). The skin over the mass appeared thin, yet there was no fluctuance or drainage observed, apart from a healed puncture wound. The mass was non-tender, and despite its disfiguring appearance, the thumb retained neurovascular integrity. Additionally, some degree of motion was preserved in the interphalangeal joint, allowing for flexion and extension movements at the interphalangeal joint. Laboratory workups included a complete blood count and comprehensive metabolic panel, which were normal. Posteroanterior and lateral radiographs of the thumb showed no evidence of trauma, calcifications, or lytic or blastic lesions (Figure 2A). An MRI without contrast of the patient’s thumb revealed an indeterminate heterogeneous exophytic mass, raising concerns for a possible soft tissue sarcoma (Figure 2B). Given this suspicion, an incisional biopsy was advised.

**Figure 3:**
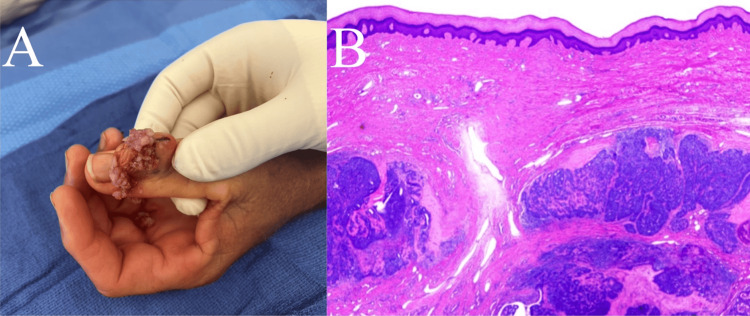
Incisional biopsy results A: Gross characteristics of the tumor were consistent with numerous fragments of friable, tan to red soft tissue aggregates; B: Low-power hematoxylin and eosin stain of the completely excised digital papillary adenocarcinoma tumor.

After the incisional biopsy was performed (Figure 3A), hematoxylin and eosin (H&E) staining and immunohistochemical analysis, including epithelial membrane antigen (EMA), p63, smooth muscle actin, SOX10, and S-100 protein, identified a biphasic tumor with both ductal and myoepithelial cells, consistent with digital papillary adenocarcinoma (Figure 3B). Cultures were also obtained and remained negative.

Given the aggressive nature of this rare lesion based on the available documented literature [2,3,4,8], our initial plan was to obtain negative margins by amputation through the proximal phalanx as well as a sentinel lymph node biopsy and subsequent follow-up with medical oncology. The patient, however, declined this treatment due to concerns about impaired hand function and asked to explore other surgical options. After a full discussion of the diagnosis, anticipated natural history of the tumor, and treatment options, he elected for wide resection and subsequent reconstruction. He verbalized an understanding of the treatment goals and the risks and benefits of this approach versus amputation. Thus, the treatment plan included two stages: a wide excision surgery to obtain negative margins, followed by a thumb reconstructive procedure to salvage thumb function. Strong recommendations were made for the patient to follow up with a medical oncologist for further evaluation and treatment, and this was organized by the surgical team for the patient. Unfortunately, the patient did not follow up on these recommendations.

**Figure 4:**
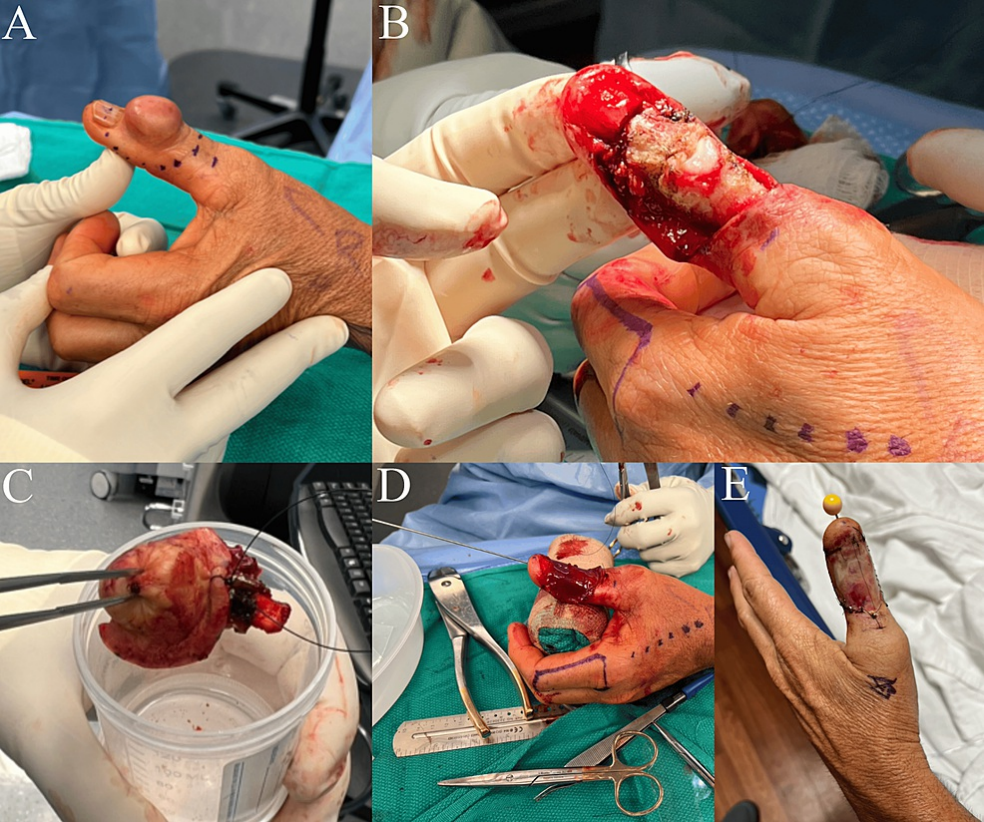
Wide excision procedure details A: Planned excision of the mass with 1 cm margins; B: Dorsal thumb after removal of the mass with complete excision of the extensor pollicis longus, dorsal capsule, and collateral ligaments; C: The excised mass with an orientation suture; D-E: Integra coverage of the dorsal thumb to prevent desiccation of the exposed phalanges, along with thumb interphalangeal joint pinning as the joint was unstable.

The second procedure was performed under general anesthesia and tourniquet control. The tumor was excised with 1 cm margins, including the proximal portion of the nail plate and matrix (Figure 4A, 4B, 4C). Intraoperative dissection revealed tortuous blood vessels surrounding the tumor, which were dissected and cauterized. The extensor pollicis longus and surrounding soft tissue were excised, along with the dorsal capsule of the interphalangeal (IP) joint and the collateral ligaments (Figure 4B). The unstable IP joint was stabilized with a 0.045 in Kirschner wire, and the defect was covered with Integra Bilayer Dermal Matrix (Integra LifeSciences, Princeton, NJ) (Figure 4D, 4E). At the follow-up visit six days later, an examination of the left thumb showed the IP joint securely pinned in extension with a clean, dry, and intact pin site. The thumb tip displayed good perfusion, and the sensation was intact along the ulnar and radial digital nerve distributions with no signs of infection. The histopathology report verified the tumor to have been completely excised with negative surgical margins.

**Figure 5:**
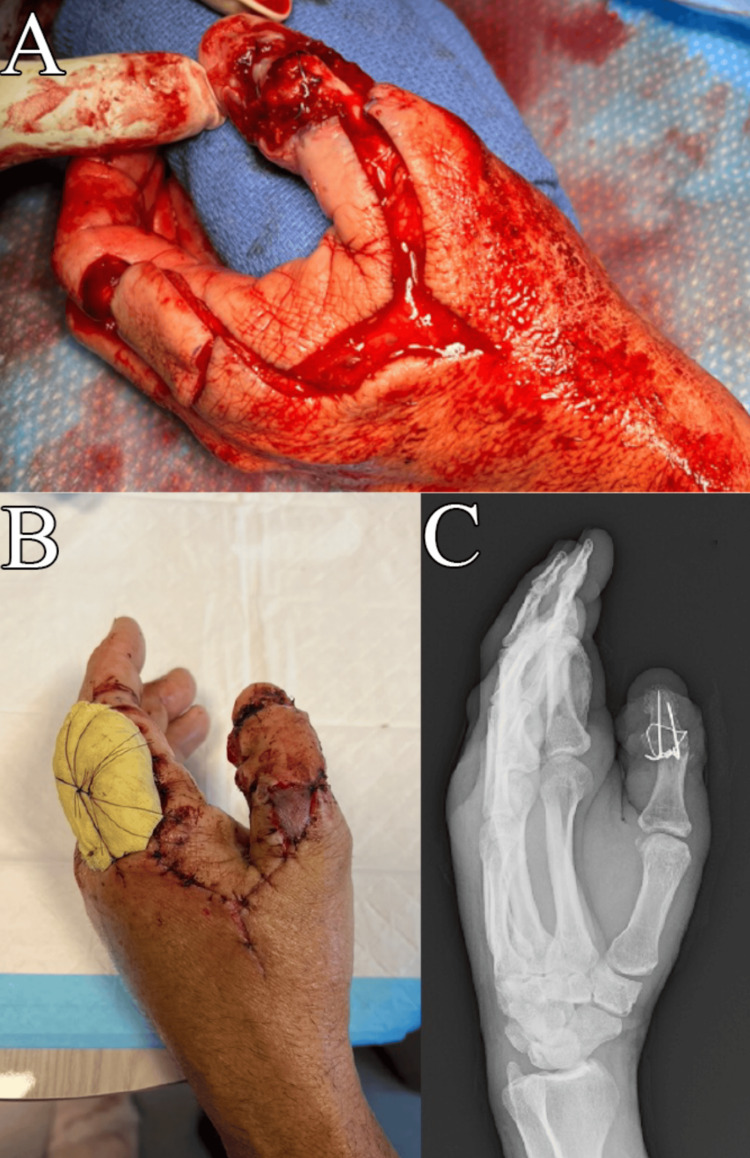
Second-stage reconstructive surgery A: Once margins were confirmed to be negative, a second-stage reconstructive surgery involving thumb interphalangeal joint fusion and FDMA flap coverage was performed; B: Bolster in place over the index finger skin graft site and a viable thumb flap three days post-operatively; C: Radiographic appearance of the fusion construct. FDMA: first dorsal metacarpal artery.

The third procedure consisted of thumb IP joint fusion dorsal skin coverage using a first dorsal metacarpal artery (FDMA) flap performed 17 days after the wide excision (Figure 5A). Removal of the Integra’s silicone layer and sutures from the previous procedure revealed no Integra incorporation over the exposed IP joint and phalanges. The FDMA flap with its pedicle was elevated, as described by Foucher and Braun [9]. A 3 x 3.5 cm donor site defect over the dorsal aspect of the index finger proximal phalanx was covered with a full-thickness skin graft harvested from the medial forearm. The IP joint was prepared for fusion at approximately 30° of flexion using K-wires and a stainless-steel cerclage wire for compression (Figure 5C). The flap was inset along the thumb defect and the hardware. A small piece of full-thickness skin graft was also placed over the pedicle to prevent excessive wound tension over the pedicle.

The patient returned for a follow-up three days later and reported well-controlled pain with no numbness or tingling. Examination showed the bolster in place over the index finger and a viable thumb flap (Figure 5B). Neurovascular function was intact in the volar aspect of the thumb. Radiographic imaging revealed appropriate fusion alignment and no hardware failure. At this point, a follow-up was planned to remove the bolster, a new thumb spica splint was placed, and the patient was advised not to use his left hand. One and a half years later, the patient continued to work in landscaping, with a functional recovery sufficient enough to engage in the physically demanding tasks that are associated with the job.

## Discussion

DPA tumors typically exhibit a histological pattern characterized by diverse papillae formations and a fusion of gland-like structures arranged back-to-back [3]. However, neither clinical nor histologic features have been identified that can reliably predict whether the tumor is prone to metastasis [3].

In the discussion of treatment options with the patient, it is crucial to consider the implications of wide local excision versus thumb amputation. Particularly for patients engaged in professions that require manual dexterity, such as musicians, artisans, or landscapers, the decision for thumb amputation can be daunting due to the resulting impairment and negative impact on their livelihood. These patients may often have a strong desire to opt out of amputation. In these cases, we recommend a treatment plan that initially involves wide local excision, ensuring negative margins, followed by reconstructive surgery aimed at preserving or restoring as much thumb function as possible. This could either be performed in a single setting or a staged fashion, as described in our report. The disadvantage of staging the procedure includes multiple procedures in the operating room. However, the benefit includes definitive histopathological verification of negative margins and greater time spent with the patient counseling and reviewing the treatment plan.

It has been demonstrated that in cases of malignant tumors of the thumb, it is possible to perform a thumb-sparing wide excision that yields good oncologic results and acceptable functional outcomes [10]. In a retrospective study involving 16 patients who underwent thumb-sparing procedures following resection for malignant tumors of the thumb or first ray with negative margins, two patients underwent reconstruction using a dorsal metacarpal artery flap, similar to our case [11]. Alternate reconstructive techniques included anterolateral thigh flaps, reverse radial forearm pedicled flaps and a toe-to-thumb transfer. The findings indicated that adhering to established principles and techniques in reconstruction can lead to satisfactory functional outcomes with minimal risk of complications. However, that study involved immediate reconstruction for the patients, whereas reconstruction in our patient was delayed until negative margins were confirmed by pathology [11]. We agree with the authors that immediate reconstruction should only be considered if the intraoperative margins are confirmed to be negative, and there is a strong likelihood that this result will be consistent with the final pathology report of the specimen [11]. Additionally, the authors recommend that prior to undertaking immediate single-stage reconstruction, it is important to consider the potential for positive margins in the final analysis and the consequent possibility of needing to amputate the thumb along with all the reconstructed tissues [11]. We ultimately selected an approach of delayed reconstruction 17 days after tumor excision, bridged by Integra coverage, which proved to be successful as far as thumb preservation and short-term thumb function are concerned.

In the case of DPA, intraoperative frozen sections may be used to verify negative margins [12], but ultimately permanent resection is a more accurate method of histopathological analysis. Although data on the use of frozen sections in DPA is scarce, a study showed that using intraoperative frozen sections (IFS) during nonmelanoma skin cancer resection led to a higher complete excision rate compared to cases without the use of IFS [13]. The rate of complete excision with IFS was 87.2%, which significantly decreased the need for additional surgeries and adjuvant therapies [13].

Amputation at the interphalangeal joint can yield an acceptable functional outcome, but for patients undergoing amputation at the metacarpophalangeal joint, it is strongly recommended to consider reconstruction techniques like a toe-thumb transfer or pollicization [10].

Sentinel lymph node biopsy is highly recommended for patients with digital papillary adenocarcinoma, as it has shown potential in predicting systemic recurrence in a small series of patients [6]. Another study revealed that 21% of patients experienced recurrence, and 5% developed metastatic disease nine years following the initial diagnosis and digit amputation [8]. Interestingly, there were no distinct morphological features to distinguish cases that recurred or metastasized from those that did not, demonstrating the challenges in predicting the evolution of this tumor. While an SLN biopsy was considered for this patient, it was ultimately not performed due to patient preferences.

Throughout the patient's treatment period with us, he was referred to an oncologist; however, he unfortunately did not pursue the recommended follow-up. In managing this condition, a comprehensive, multidisciplinary strategy is preferred. This approach should involve collaboration between medical oncology specialists, orthopedic oncology, and a hand or plastic surgeon familiar with soft tissue reconstruction in the hand. Patient education is crucial, ensuring patients are fully informed about their treatment options, potential outcomes, and the importance of ongoing surveillance. Regular patient follow-up is important to monitor for recurrence and manage any post-surgical complications or rehabilitation needs effectively.

## Conclusions

When managing a case of DPA, we emphasize the importance of comprehensive surgical and oncological management. Delayed reconstructive surgery following wide excision is feasible after a thorough margin assessment, allowing for an effective functional recovery. This case report underscores the viability of a thumb-sparing approach, even in the context of aggressive tumors like DPA, where traditional management might favor more radical procedures such as amputation. Our case management differed from previously published case reports by prioritizing thumb preservation and functionality through a staged reconstruction approach. This approach not only provided a favorable oncologic outcome but also ensured that the patient could return to his physically demanding job without significant disability.
